# Re-Examining the Financial Structure and Health Nexus in Asian Economies

**DOI:** 10.3389/fpubh.2022.860325

**Published:** 2022-03-04

**Authors:** Yanling Xu, Ran Tao, Chaudhry Kashif Mahmood, Mehmet Altuntaş

**Affiliations:** ^1^School of Business, Wuchang University of Technology, Wuhan, China; ^2^Qingdao Municipal Center for Disease Control and Prevention, Qingdao, China; ^3^College of Business Administration, Imam Abdulrahman Bin Faisal University, Dammam, Saudi Arabia; ^4^Department of Economics, Faculty of Economics, Administrative and Social Sciences, Nisantasi University, Istanbul, Turkey

**Keywords:** financial structure, GDP, life expectancy, health, Asia economies

## Abstract

The study's main purpose is to estimate the impact of the financial structure of Asian economies on the healthcare sector from 2000 to 2019. For empirical estimation, we relied on two-stage least square (2SLS) and generalized method of moment (GMM) estimation techniques. Two different proxies, infant mortality and life expectancy, were used in the analysis to represent the health status of the people. The findings of both 2SLS and GMM models confirm that improved financial structure causes life expectancy to rise and infant mortality to fall. Moreover, the increased usage of the internet also exerts a positive impact on the health status of Asians. Further, the rise in gross domestic product (GDP) and health expenditures also improve the health status of Asians by increasing their life expectancy and reducing their infant mortality rate. Improvement in financial structure causes the health status of the people to rise. Therefore, to achieve superior health status, the development of financial structure should be part and parcel of health policies and strategies in Asian economies.

## Introduction

The impact of financial structure on economic development has been explored quite extensively in the literature, but very little work is done on the nexus between financial structure and poverty (1). It is argued that poverty is the root cause behind several socio-economic issues, such as food insecurity, malnutrition, and poor health, in developing and less developed economies. Most specifically, some studies tried to explore the contribution of financial structure on poverty reduction through banking or markets-based channels of financial intermediation (2). The existing literature claims that lack of finance is the main determinant of poor health outcomes and persistent poverty (3).

The literature reports that financial structure could affect health outcomes through various channels. First, it claims that shortage of finance could be a major cause behind the poor health of individuals (4). Due to the high per-unit cost of lending and other limitations, individuals cannot borrow money for their health needs. It further highlights that easy access to financial services makes it convenient for households and entrepreneurs to enhance their economic opportunities and manage risks (5, 6). Moreover, the financial structure also influences the economic outcomes and opportunities of individuals without affecting their consumption of financial amenities (7, 8). For instance, economic activities increase due to an expansion in the financial structure that increases labor demand. Due to employment generation and increased economic growth, people's incomes rise, enabling them to get better healthcare services. A bulk of literature reports that financial structure could improve the health outcomes of the economies through the channels of economic growth, employment generation, and poverty alleviation (9, 10).

Merton (11) states that a financial structure provides services, such as a payment system, a funds pooling mechanism, an easy source for transferring funds across time and space, risk control and uncertainty management, and management for asymmetric information. These services can be performed in diverse methods in various economies. The question under investigation is: how do corporations manage and raise funds? Large organizations usually raise finance through the banking sector and public markets. The financial structure of an economy consists of financial technology and institutions that define how financial activities take place at some specific time (12). Different institutions also perform similar kinds of functions under different kinds of rules. For example, U.S. and Japan have completely different financial structures at similar levels of economic growth (13). Hence, it can be stated that financial structure is determined within the system (14). A bulk of research has explored the relationship between economic growth and financial structure (15–17). In the existing stock literature, indicators to measure financial structure include a proportion of funds generated externally by organizations, stock market capitalization as a share of gross domestic product (GDP), stock market trading as a ratio of GDP, stock market turnover, etc. (18). A financial structure can promote or hinder the country's economic development that influences the income status of households (19), thus directly or indirectly influencing the health sector of the economy.

A financial structure may influence life expectancy through several channels. A financial structure positively influences health quality since easy access to finance helps households make better and healthy choices about food, treatment, accommodation, and overall living styles. Similarly, the financial structure may enhance life expectancy *via* increasing GDP *per capita*, gender equality, infrastructure, and education (20). A bulk of studies argue that financial structure development enhances economic development and alleviates poverty through mobilizing savings, promoting innovations, resource allocation in the production sector, and reducing the cost of the transaction, thus improving the quality of life and health outcomes (21, 22). Consequently, several studies have confirmed these arguments in both multi-country and single-country contexts (23). Studies showed that financial structure and economic growth are positively related, thus improving health quality as a higher income helps in the provision of good quality food, health care facilities, and housing (24).

In the present era, after the coronavirus disease 2019 (COVID-19) pandemic, most specifically, health challenges have evolved throughout the world. Thus, the cost of innovation in the health sector has increased significantly. Government budgets are in deficit, savings rates decrease, and private, and public sectors wonder how healthcare costs can be fulfilled under this continuously increasing universal health coverage (UHC). It is observed that the financial sector, especially investors from insurance agencies and asset management companies, can provide long-term loans under these circumstances (25). International calls on UHC have directed many economies to implement reforms in the health sector. However, due to the COVID-19 pandemic, economic development has declined in middle-income and lower-income economies (26). In the prevailing scenario, international agencies emphasize the significance of financial structure for the health sector. Chireshe and Ocran (27) state that financial structure development is fundamental for financing the health care sector as it provides opportunities for the resource mobilization of government, private firms, and households to finance the expenditures on health care. A well-organized financial structure is proficient in resource mobilizing, which is required to finance long-term investments and current health-related expenditure, compared to less developed financial markets (28). Well-managed financial structures expand government budgets in the health sector by enlarging tax revenues (29, 30).

There exists a plethora of empirical and theoretical studies investigating the impact of financial structure on several indicators such as economic growth, FDI, energy consumption, employment, factor accumulation, and productivity improvement (26). However, limited studies have considered the impact of financial structure on the health sector. To fill this lacuna, we explore the nexus between financial structure and health outcomes in China (31). In this study, financial structure is measured by two proxy measures: stock market capitalization and stock market turnover (32). Health outcome is measured by infant mortality rate and life expectancy. The study employs the Generalized Method of Moment (GMM), and two-stage least square (2SLS) approaches for empirical investigation from 1990 to 2019. The study contributes the following: First, it provides an extensive evaluation of the redistribution of financial resources in the health sector (33). Second, this study will help achieve the health-related target of Sustainable Development Goals (SDGs) through financial structure development in China (34). Third, this study will help policymakers design policies that pave the path toward a speedy recovery from the COVID-19 pandemic (35). Fourth, it highlights the important implication of the financial structure on the health quality of Asian economies. The findings of this study will help policymakers and decision-makers in adopting suitable methods for the development of the financial sector and the improvement of health quality.

## Model and Methods

Financial sectors play a vital role in the process of capital formation. Meanwhile, different financial sectors have different mechanisms affecting the mobilization of capital allocation, savings, and risk management and thus, their effects on poverty (3). A well-functioning financial structure not only alleviates poverty and relaxes the problem of income distribution by creating job opportunities for the poor, it also improves health outcomes (36, 37). To detect the influences of financial structure on health, we have employed the following model forms:


$$\begin{array}{l} \text{Healt}{\text{h}}_{\text{it}}=\;\pi_{0}+\;\pi_{1}\text{F}{\text{S}}_{\text{it}}+\pi_{2}\text{Interne}{\text{t}}_{\text{it}}+\pi_{3}\text{GD}{\text{P}}_{\text{it}}+\\ \;\pi_{4}\text{H}{\text{E}}_{\text{it}}+\pi_{5}\text{Trad}{\text{e}}_{\text{it}}+\alpha_{\text{i}}+\varepsilon_{\text{it}} \end{array}$$

where the health outcomes depend on financial structure (*FS*), internet users (internet), GDP *per capita* (GDP), health expenditure (HE), and trade openness (Trade). A financial structure improves health outcomes by reducing poverty; thus, estimates of π_1_ is expected to be positive in the equation. As for the impacts of our control variables, internet users, GDP, health expenditure, and trade openness could positively affect health. In equation (1), π_1_, π_2_, π_3_, π_4_, and π_5_ represent the elasticities of financial structure, internet users, GDP, health expenditure, and trade openness, respectively. Previous standard literature (38–40) noted that digitalization, economic development, health expenditure, and trade openness improve human health and well-being by reducing income inequity and poverty. Thus, we introduce the control variables (digitalization, economic development, health expenditure, and trade openness) into the health model to study the evolution of the effect of financial structure variables. In equation (1), α_*i*_ is an unobserved country-specific effect while ε_*it*_ is the error term, and it is assumed that this term is independent and identically distributed (*i.i.d*.). In equation (2), *Health*_*it*−1_ is the lagged level of health outcomes. The augmented panel model is:


$$\begin{array}{l} \text{Healt}{\text{h}}_{\text{it}}=\;\pi_{0}+\lambda_{1}\text{Healt}{\text{h}}_{\text{it}-1}+\;\pi_{1}\text{F}{\text{S}}_{\text{it}}+\pi_{2}\text{Interne}{\text{t}}_{\text{it}}+\\ \;\pi_{3}\text{GD}{\text{P}}_{\text{it}}+\pi_{4}\text{H}{\text{E}}_{\text{it}}+\pi_{5}\text{Trad}{\text{e}}_{\text{it}}+\alpha_{\text{i}}+\varepsilon_{\text{it}} \end{array}$$

The financial structure is the endogenous regressor and main variable in our augmented dynamics panel models.

The endogeneity problem is likely to occur following the presence of correlation between the independent variable/s and error term. To account for endogeneity in the fundamental model, we employ the 2SLS and GMM. Thus, we estimate the econometric model with an endogenous variable using the 2SLS, which fixed the problem of endogeneity in the panel model. The second method is panel model via GMM which is used to examine the impact of the financial structure on health. With the estimator of GMM, we also correct for the possible autocorrelation and heteroskedasticity in the error structure by using a reliable estimator. A traditional Sargan diagnostic test is carried out to ensure the validity of the GMM estimator. The choice of good instruments is crucial to determine a better outcome. Both types of panel instrumental variables estimation approaches are good, but GMM is a more valuable approach than 2SLS in panel data. Such a method is a more useful choice when the time period (t) is smaller, as in our case (2000 to 2019). Normally, two types of the GMM estimator exist, namely, two-step and one-step GMM. Regarding the econometric problems, the two-step estimator attains better results than the one-step estimator (41). Thus, we employed a two-step GMM estimator in our panel model.

## Data

The study re-examines the relationship between financial structure and health outcomes in Asian highly populated economies. These economies include China, India, Indonesia, Iran, Korea Rep., Japan, Russia, Saudi Arabia, Thailand, and Turkey. The study used time-series data from 2000 to 2019. Detailed information about statistical inferences, definitions, symbols, and sources of data are given in Table 1. The study used two proxies to measure health outcomes, namely infant mortality rate and life expectancy rate. Financial infrastructure is also measured using two proxies: stock market capitalization (in percent of GDP) and stock market turnover ratio (in percent). Internet users (in percent of the population), *per capita* GDP (constant 2010 US$), current health expenditures (in percent of GDP), and trade (in percent of GDP) are used as control variables. The data for all variables are obtained from the World Bank and IMF, while Table 2 provided the results of the correlation matrix and reported that the model is free from the multicollinearity problem.

**Table 1:** Data descriptions and sources.

**Variable**	**Mean**	**Std. dev**.	**Min**	**Max**	**Definitions**	**Sources**
IM	16.75	13.66	1.800	66.70	Mortality rate, infant (per 1,000 live births)	World bank
LE	73.64	5.146	62.50	84.35	Life expectancy at birth, total (years)	World bank
FSS	3.829	0.600	1.885	4.971	Stock market capitalization to GDP (%)	IMF
FSA	4.337	0.860	1.152	6.322	Stock market turnover ratio (%)	IMF
Internet	3.108	1.235	−0.640	4.714	Individuals using the Internet (% of population)	World bank
GDP	8.983	1.027	6.717	10.80	GDP per capita (constant 2010 US$)	World bank
HE	5.007	1.975	1.909	11.70	Current health expenditure (%of GDP)	World bank
Trade	60.52	27.58	19.56	140.4	Trade (%of GDP)	World bank

**Table 2:** Correlation matrix.

	**IM**	**LE**	**FSS**	**FSA**	**Internet**	**GDP**	**HE**	**Trade**
IM	1							
LE	−0.792	1						
FSS	−0.314	0.376	1					
FSA	−0.146	0.326	0.391	1				
Internet	−0.823	0.771	0.462	0.214	1			
GDP	−0.855	0.811	0.304	0.251	0.722	1		
HE	−0.521	0.698	0.174	0.052	0.515	0.668	1	
Trade	−0.208	0.040	0.254	0.098	0.058	−0.019	−0.364	1

## Results and Discussion

Table 3 provides the empirical findings of the financial infrastructure and infant mortality nexus, while displaying the empirical findings of financial structure and life expectancy nexus. The study used 2SLS and GMM approaches for empirical investigation. In columns 1 and 3, stock market capitalization is used as a focused variable, while in columns 2 and 4, stock market turnover is used as a focused variable in both tables. In the case of stock market capitalization, it is found that stock market capitalization impact on infant mortality is significant and negative at a 1% level in both models. It shows that stock market capitalization results in reducing infant mortality rates in highly populated economies. Results show that a 1% increase in stock market capitalization reduces infant mortality by 0.644% in the 2SLS model and 0.523% in the GMM model. Findings of control variables display that internet, health expenditure, and GDP impact on infant mortality rate is significant and only negative in the GMM model.

**Table 3:** Effects of financial structure on infant mortality.

	**2SLS**	**2SLS**	**GMM**	**GMM**
	**(1)**	**(2)**	**(3)**	**(4)**
L.infant mortality			0.996^***^	1.003^***^
			(0.011)	(0.011)
FSS	−0.644^***^		−0.523^***^	
	(0.004)		(0.003)	
FSA		−0.715^***^		−0.651^***^
		(0.243)		(0.122)
Internet	−0.048	−0.041	−0.005^**^	−0.004^**^
	(0.049)	(0.055)	(0.002)	(0.002)
GDP	−0.464	−0.505^**^	−0.371^***^	−0.396^***^
	(0.630)	(0.202)	(0.121)	(0.119)
HE	−0.285	−0.083^***^	−0.005^***^	−0.004^***^
	(0.960)	(0.028)	(0.001)	(0.001)
Trade	0.050	0.002	0.015	0.014
	(0.134)	(0.012)	(0.014)	(0.025)
year			−0.003^***^	−0.004^***^
			(0.001)	(0.001)
Constant	−4.319	−8.453^***^	−10.31	−11.15
	(1.232)	(1.497)	(1.382)	(1.432)
Observations	200	200	180	180
Number of code	10	10	10	10
Sargan test			0.562	0.875

*** *p < 0.01;*

** *p < 0.05*.

In the case of stock market turnover, it is found that the stock market turnover effect on infant mortality rate is significant and negative at 1% level of significance. It reveals that a 1% upsurge in stock market turnover decreases infant mortality rate by 0.715% in 2SLS model and 0.651% in GMM model. The findings confirm that an expansion in financial structure either in the form of stock market capitalization or stock market turnover reduces infant mortality rate significantly, thus suggesting that governments of these economies can opt for financial expansion policies to improve the health outcomes in the region. The world has to face manifold challenges related to health financing because its health burden has changed. Financial sectors such as investors from insurance companies, asset management companies, and banks play a significant role in providing long-term finance for the health industry. The financial sector plays manifold roles such as lenders, investors, and intermediaries for equity and debt investments from the capital to equity markets. The development of the financial sector contributes to facilitating innovation, economic growth, entrepreneurship, and immensely supports the country's health sector. Although an optimal level of financial structure provides loans for investment, risk management services, and liquidity to fill financial gaps, some current evidence also show that the financial structure has a favorable impact on the health industry (42). The nexus of the financial structure and health system has a predominant impact in post-COVID recovery not only in developed economies but also in developing economies as well. A similar finding is also found by Sahay et al. (43). This finding is also backed by Brei et al. (44), who noted that financial structure helps reduce income inequality and poverty, which in turn improves human health in developing countries. The findings also infer that financial structure improves human health *via* income and wealth channels.

A financial sector development can help people through many channels. Studies claimed that lack of finance is the major cause behind the poor health of people (45). Poor people are less capable of borrowing money because of the high cost of lending from financial institutions. Our finding is also supported by Claessens and Feijen (46), who claimed that financial sector development in the health sector could directly affect households' economic outcomes and opportunities without affecting their consumption of financial services. The results suggest that the good structure of a financial system enhances economic activities that increase labor demand, which increases the income level of households and thus, increases their capacity to obtain health services. Moreover, a good financial structure improves healthcare services by reducing poverty (3). Finally, the findings of control variables display that GDP and health expenditure impact on infant mortality rate is significant and negative in both models while the impact of the internet is significant and negative only in the GMM model.

In Table 4, stock market turnover and stock market capitalization's impact on life expectancy has been explored by using 2SLS and GMM approaches. The findings reveal that the stock market capitalization effect on life expectancy is positive and significant at a 5% level in the 2SLS model and a 1% level in the GMM model. It shows that a 1% expansion in stock market capitalization enhances life expectancy by 0.964% in the 2SLS model and 0.752% in the GMM model. All control variables are statistically insignificant in the case of the 2SLS model, while the internet and GDP display significant and positive impacts on life expectancy in the GMM model. The findings for stock market turnover again confirm a significant and positive impact on life expectancy at a 1% level in both 2SLS and GMM models. It infers that a 1% increment in stock market turnover raises life expectancy by 0.085% in the 2SLS model and 0.091% in the GMM model. These findings again confirm that stock market capitalization and stock market turnover result in improving the health outcome in highly populated Asian economies by increasing the life expectancy of individuals. However, findings show that stock market capitalization plays a dominant role in determining health outcomes compared with stock market turnover. In the case of control variables, GDP and health expenditures produce a significant and positive impact on life expectancy in the 2SLS model. In the GMM model, findings reveal that the internet and GDP produce a significant and positive impact on life expectancy.

**Table 4:** Effects of financial structure on life expectancy.

	**2SLS**	**2SLS**	**GMM**	**GMM**
	**(1)**	**(2)**	**(3)**	**(4)**
L.life expectancy			0.959^***^	0.959^***^
			(0.019)	(0.020)
FSS	0.964^**^		0.752^***^	
	(0.464)		(0.234)	
FSA		0.085^***^		0.091^***^
		(0.025)		(0.027)
Internet	0.079	0.003	0.049^*^	0.058^**^
	(0.244)	(0.006)	(0.029)	(0.029)
GDP	0.445	0.381^***^	0.373^***^	0.343^***^
	(0.365)	(0.021)	(0.095)	(0.101)
HE	0.039	0.004^**^	0.027	0.022
	(0.114)	(0.002)	(0.019)	(0.019)
Trade	0.006	0.007	0.002	0.001
	(0.016)	(0.014)	(0.001)	(0.001)
Year			0.018	0.017
			(0.010)	(0.010)
Constant	5.412	3.881^***^	8.170^***^	7.746^***^
	(4.312)	(0.155)	(1.650)	(1.785)
Observations	200	200	180	180
Number of code	10	10	10	10
Sargan test			0.801	0.504

*** *p < 0.01;*

** *p < 0.05;*

* *p < 0.1*.

## Conclusions and Implications

The study's main purpose is to estimate the impact of financial structure on the healthcare sector of Asian economies. The correlation matrix confirms that perfect collinearity does not exist between any pair of two variables; hence, we can proceed to our analysis. Our data set is longitudinal in nature, composed of cross-sections and time series observations. However, the time series is not long enough; therefore, we have recourse to traditional panel data techniques such as 2SLS and GMM. Two different variables, infant mortality and life expectancy, represent the health status of the people living in Asian economies. From the infant mortality model estimates, we observed a negative association between stock market capitalization and stock market turnover ratio with both 2SLS and GMM techniques, confirming that improvement in financial structure reduces infant mortality rate in Asian economies. Similarly, the estimated coefficients of the internet also appeared to be significantly negative in the infant mortality model in only the GMM model, implying that an increase in the usage of the internet helps reduce infant mortality in Asian economies. Likewise, the rise in GDP and health expenditures also causes the infant mortality rate to fall irrespective of the estimation technique.

Moreover, in the life expectancy model, the relationship between life expectancy and stock market capitalization and stock market turnover ratio is positive and significant with both 2SLS and GMM estimation techniques. From these findings, we can confer that the development of financial structure in Asian economies causes life expectancy to rise significantly. Once again, the internet estimate turns out to be significant and positive with the GMM estimation technique, implying that a rise in internet usage helps increase life expectancy. On the other side, estimated coefficients of GDP and health expenditures, in both 2SLS and GMM models, turn out to be positively significant.

### Policy Implications and Limitations

These results are essential for policymakers. Improvement in financial structure causes the health status of people to rise by increasing life expectancy and reducing infant mortality. Therefore, policymakers should focus on developing the financial structure by increasing stock market capitalization and stock market turnover ratio. Moreover, to achieve superior health status development, the financial structure should be part and parcel of health policies and strategies in Asian economies. The accountability of financial institutions promotes a better understanding of the complexities of association between financial structure and health outcomes; thus, the improvement of the quality of financial structure is suggested. Financial structure develops under stronger property rights; thus, such reforms are required in a financial structure to help improve human health. Further, the increased internet usage causes life expectancy to increase and infant mortality to fall. Therefore, the use of the internet should be increased in the health sector. The Internet can be used to disseminate health-related information at a great speed. Even people from far-flung areas can use the internet to consult a doctor and read the latest research articles on a health topic.

The study ignores the financial crisis, health infrastructure, and health education in empirical analysis. Future studies should include financial crisis variables in the analysis and use other measures of human health such as mental health, maternal health, and other chronic diseases. Future research can incorporate the role of financial globalization in modeling. Moreover, they can explore the asymmetric relation between health and financial structure by adopting advanced econometric approaches. Future researchers can explore this nexus by considering a sample of developing and developed economies to compare as the health infrastructure, health education, economic development, and health-related facilities of developed economies are different from developing ones.

## Data Availability Statement

Publicly available datasets were analyzed in this study. This data can be found at: https://data.worldbank.org/.

## Author Contributions

YX and RT: conceptualization, software, data curation, and writing—original draft preparation. CM: methodology and writing—reviewing and editing. MA: visualization and investigation. All authors contributed to the article and approved the submitted version.

## Conflict of Interest

The authors declare that the research was conducted in the absence of any commercial or financial relationships that could be construed as a potential conflict of interest.

## Publisher's Note

All claims expressed in this article are solely those of the authors and do not necessarily represent those of their affiliated organizations, or those of the publisher, the editors and the reviewers. Any product that may be evaluated in this article, or claim that may be made by its manufacturer, is not guaranteed or endorsed by the publisher.
